# The Cost of Treating Advanced Non-Small Cell Lung Cancer: Estimates from the Chinese Experience

**DOI:** 10.1371/journal.pone.0048323

**Published:** 2012-10-31

**Authors:** Xiaohui Zeng, Jonathan Karnon, Siying Wang, Bin Wu, Xiaomin Wan, Liubao Peng

**Affiliations:** 1 Department of Pharmacy, the Second Xiangya Hospital of Central South University, Changsha Hunan, People’s Republic of China; 2 School of Pharmaceutical Sciences, Central South University, Changsha Hunan, PR China; 3 Department of Public Health, University of Adelaide, Adelaide, South Australia, Australia; 4 Department of Pharmacy, School of Medicine, Shanghai Jiaotong University, Renji Hospital, Shanghai, People’s Republic of China; University of Porto, Portugal

## Abstract

**Background:**

Because of the potentially significant economic burden of healthcare costs associated with many diseases, it is critical that regulatory and medical insurance organisations collect and utilise data on the cost-effectiveness of care provision to make rational policy decisions. However, little is known about healthcare costs in China.

**Methodology/Principal Findings:**

Based on health expenditure data for 253 cases of advanced non-small cell lung cancer (NSCLC) registered at the Second Xiangya Hospital of Central South University in China between 2006 and 2010, the cost of care provision was analysed. The monthly and aggregate annual medical costs were estimated for patients who were in either a progression-free state (PFS) or a disease-progression state (DPS). Monthly healthcare costs accumulated during the terminal 3 months were collected separately. The mean cost of treatment for PFS and DPS patients over one year was approximately US$11,566 and $14,519, respectively. The monthly costs for all patients were higher initially than in the subsequent months (PFS: $2,490; DPS: $2,503). For PFS patients, healthcare expenditures stabilised after the 7th month, with a mean monthly medical expenditure of $82.49. For DPS patients, expenditures stabilised after the 9th month, and the mean expenditure during the 9th month was $307.9. Medical care costs in the three successive months prior to death were $3,754, $5,829 and $7,372, respectively.

**Conclusions/Significance:**

The economic evaluation of health care technologies is becoming ever more important in China, especially in disease areas for which new and expensive therapies are being introduced on a regular basis. This is first paper to present empirically estimated China-specific costs associated with the treatment of NSCLC. The cost estimates are presented in a format that is specifically intended to inform cost-effectiveness analyses of treatments for NSCLC, and hence, contribute to the more efficient allocation of limited healthcare resources in China.

## Introduction

Lung cancer, the most commonly diagnosed form of cancer, is also the deadliest form of cancer in males [1]. There has been a continuous decline in lung cancer death rates in most Western countries [2]. However, in developing countries, such as China and other Asian, and African countries, the rates are increasing [2]. Consequently, lung cancer is becoming an increasingly significant public health problem in China [3]. Non-small cell lung cancer (NSCLC) accounts for more than 85% of all lung cancer cases [4], approximately 40% of which are first diagnosed at the advanced stage of malignancy [5]. Healthcare spending for NSCLC has increased because of the growing number of new therapies [6], including third-generation chemotherapies and molecular targeted drugs (angiogenesis inhibitors, tyrosine-kinase inhibitors, and pemetrexed, a folate antimetabolite) [5], [7], [8].

Because of the significant economic burden that patients often bear as a result of their healthcare costs, it is critical that regulatory and medical insurance organisations collect and utilise data to estimate the cost-effectiveness of alternative options for the provision of health care [9]. Mathematical models that can estimate the long-term cost-effectiveness of alternative strategies using available data, while making some necessary and appropriate assumptions are now commonly utilised to guide policy decision-making [10], [11]. Such models comprise health states, through which a cohort of hypothetical patients may progress over the remainder of their modelled lifetime. However, to populate the model parameters associated with the time spent in each of the defined model states requires data on patients’ health status over time and associated data on healthcare expenditures and quality of life [12]. Unfortunately, these parameters are often difficult to estimate due to a lack of available and current data, especially in China, a developing country where the field of pharmacoeconomics remains in its infancy [13].

**Figure 1:**
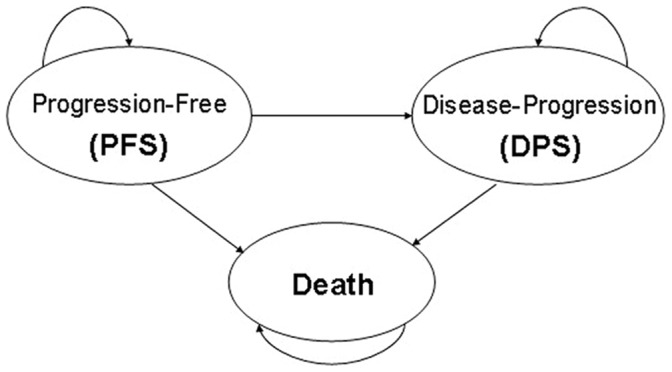
Markov process of advanced NSCLC.

The aim of this research, from the perspective of the Chinese healthcare system, was to inform patients and oncologists about the healthcare costs associated with treating advanced NSCLC, including more specialised knowledge about the time- and health status-related medical costs that patients may anticipate. Figure 1 shows the Markov processes used by Carlson JJ et al [14] and Wu B et al [15], [16], in whom progression-free state (PFS) denotes a steady state of disease without signs of worsening and disease-progression state (DPS) signifies progression of the disease. Our study had three specific objectives: first, to calculate the total costs over a 12-month follow-up period for patients in a progression-free state (PFS) and a disease-progression state (DPS); second, to estimate the monthly costs following a PFS and PDS; and last, to estimate average healthcare expenditures during the terminal phase of the disease.

## Materials and Methods

### Data Collection

To conduct the analysis, two databases were designed and created at the Second Xiangya Hospital of Central South University in China. The databases were populated from the medical records of 253 patients who were diagnosed with advanced NSCLC.

**Figure 2:**
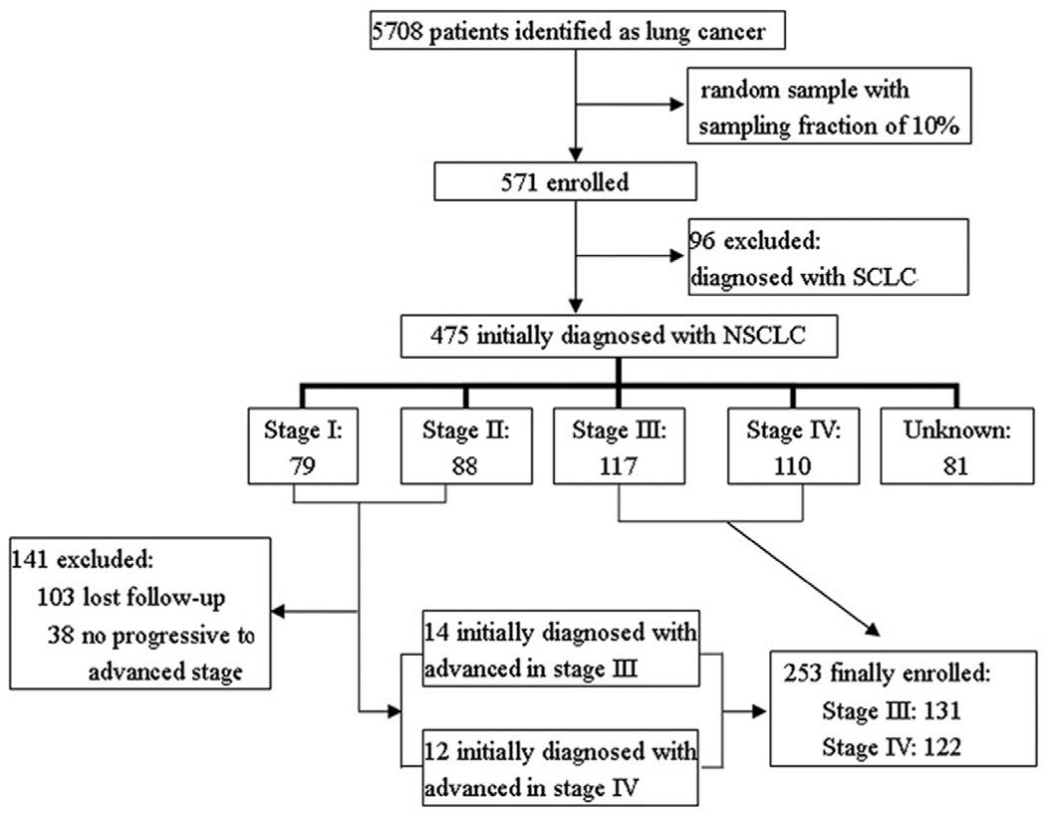
Flow-chat of patients enrolled.

A total of 5,708 patients were diagnosed with lung cancer in the Second Xiangya Hospital’s oncology department records from 2006 to 2010. A simple random sample (n = 571), with a sampling fraction of 10%, was generated online using an automated random number generator (http://stattrek.com/Tables/Random.aspx). A total of 475 of the randomly selected patients had been diagnosed with NSCLC (the rest were SCLC), and only patients in the advanced stage were included. Some patients who had been diagnosed at stage I or II but who had then progressed to an advanced stage were also included. Ultimately, 253 patients met the inclusion criteria for our study (Figure 2). We screened all 253 of the eligible patients, including both inpatients and outpatients. The survival time for each was split into PFS and DPS (where relevant), and data for PFS and DPS were added to two separate databases. The PFS database contained 228 patients, and the DPS database contained 104 patients. We populated the two databases as thoroughly as possible using case records from the medical record library and the electronic medical record system, medical reports from the information section (a department in the hospital), and the Second Xiangya Hospital oncology department’s outpatient database. Using the patient medical information contained in the medical record library and the electronic medical records system, all of the basic health and disease management information for each patient was recorded on to the relevant database, i.e. PFS or DPS, according to patient status. Through medical reports from the information section and the oncology outpatient database, we collected data on each patient’s use of all healthcare-related resources from the date of diagnosis to the date of death or his/her last clinic appointment with the oncology department. Data on resource utilisation were collected for a maximum of 12 months for each patient.

To estimate the monthly costs associated with PFS and DPS, within each of the two databases, each patient’s pattern of resource use was divided into different intervals by month in chronological order. Monthly and annual observations were only included if a patient remained in the same state (PFS or DPS) for the full month or year, respectively. Expenditures on healthcare resources that were used for more than a month (in the inpatient setting, for example) were averaged over the total number of days utilised, and that average value was used to calculate the mean monthly costs. For example, one patient who was hospitalised for 40 days over a 2-month period spent 25 days of 1 month and 15 days of the subsequent month receiving treatment. Thus, the first month’s expenditures were calculated as 25/40 of the total cost of hospitalisation, and the surplus (15/40) was added to the next month’s tally.

Resource use distinguished between those incurred in inpatient and outpatient settings. Inpatient costs reflected the cost of hospitalisation and other related expenses, including the costs for medicine, scans, biopsies, therapy, radiation, nursing care, materials and ward bed. We included any outpatient costs associated with adverse reactions and/or re-examinations.

Unit costs in this study were estimated in US dollars (USD), corresponding to the 2010 consumer price index and assuming an average exchange rate of 1 USD to 6.7695 Renminbi (RMB). The majority of the unit costs were derived from the Publicity Medicine Prices of Hunan [17], which are based on data collected from all of the hospitals in the Hunan province of China. Unit costs that could not be obtained from the Publicity Medicine Prices of Hunan were collected from the Second Xiangya Hospital. The unit cost of radiotherapy reflects the radiation fee for a single-fraction irradiation session. Table 1 presents a selection of unit costs.

This study was approved in accordance with the principles of the ethics committee in the Second Xiangya Hospital of Central South University (Changsha, People’s Republic of China). Written consent was given by the patients for their information to be stored in the hospital database and used for research.

### Statistical Analysis

Three methods of analysis are described in the following section.

The first analysis estimates the total aggregate healthcare costs over one year for patients with advanced NSCLC. The mean costs were estimated and evaluated using 95% confidence intervals (CIs) through bootstrapping with the R software (version 2.14.0; R Development Core Team, Vienna, Austria).

The second analysis estimated the average monthly costs for PFS and DPS patients. The purpose of this analysis was to calculate the sequential average monthly costs for PFS and DPS patients. The mean monthly costs over the first year following entry to each state were estimated, and 95% CIs were generated through a bootstrapping method.

The final analysis estimated the end-of-life healthcare costs for 63 patients. Patient health expenditures during the terminal phase, also referred to as the end-of-life costs, are different from the costs attributable to standard therapy [18]. For the 63 patients with advanced NSCLC who died during the study period, costs incurred during their last 3 months of life were collected and analysed to estimate the mean end-of-life costs, with corresponding bootstrapped CIs.

## Results

In total, 253 eligible cases were identified and assessed, and they were classified by their PFS, DPS or terminal-stage status. The study included 228 PFS patients, 104 DPS patients and 63 terminal phase patients.

### Patient Characteristics

**Figure 3:**
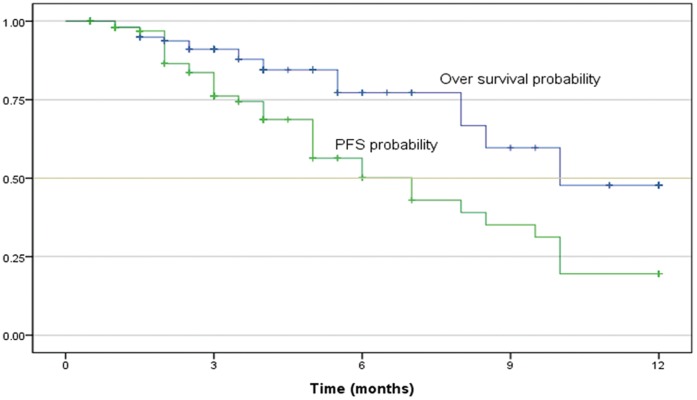
Progression-free survival and overall survival of patients.

Table 2 shows demographic information for all of the NSCLC patients in the random sample of advanced-stage patients. The mean and median ages of the 253 patients were 58 and 59 years, respectively, and the median survival time and progression-free survival time of the patients were 10.5 and 7.0 months, respectively. Figure 3 shows Kaplan-Meier curves for the overall survival and progression-free survival of the enrolled patients.

**Table 1:** Unit cost of selections.

Resource	Unit cost (US $)
Examination	
CT chest scan	59
Enhanced CT chest	96
Ultrasonic B abdomen	49
MRI brain	134
SPECT	87
Radiotherapy	
Radiation (common)	21
Three dimensional conformal RT (3DCRT)	30
Intensity Modulated RT (IMRT)	44
Chemotherapy	
1 Course of TP treatment (270 mg,120 mg)	1,738
1 Course of DP treatment (120 mg,120 mg)	1,521
1 Course of GP treatment (3.6 g,120 mg)	1,632
1 Course of NP treatment (80 mg,120 mg)	716
Docetaxel (20 mg)	97
Molecular targeted therapy	
Pemetrexed (200 mg)	321
Gefitinib (250 mg)	81
Erlotinib (150 mg)	95
rh endostatin (15 mg)	157

TP: paclitaxel plus cisplatin; GP: gemcitabine plus cisplatin; DP: docetaxel plus cisplatin; NP: vinorelbine plus cisplatin.

**Table 2:** Patient characteristics.

	Total patients	Advanced NSCLC patients
Number	475	253
Male/female	337/138	182/71
Age(years)		
Mean	59	58
Median	59	59
Range	31–84	32–81
Tumour stage		
Stage I	79	–
Stage II	88	–
Stage III	117	131
Stage IV	110	122
Not known	81	–
Overall survival time (months)		
Median	–	10.0
Mean	–	9.1
PFS survival time (months)		
Median	–	6.0
Mean	–	6.5

The cost results are described in the following sections and include the 1-year average costs, the monthly costs for PFS and DPS patients, and terminal-phase expenditures. The results are depicted with error bars, and the high and low means are presented with 95% CIs (with the Sigma Plot Scientific Graphing Software version 10.0; Jandel Scientific, San Rafael, CA, USA).

### Total 1-year Costs

**Figure 4:**
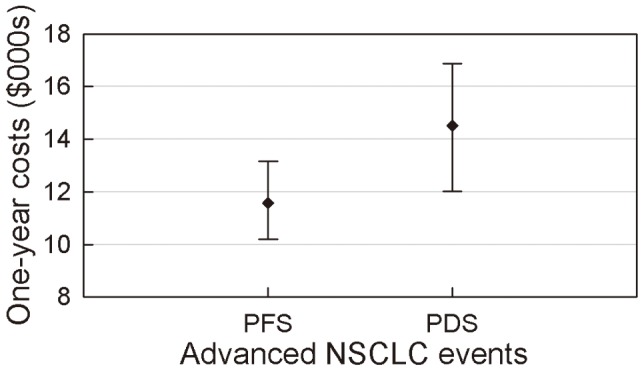
One-year costs (with 95%CI) of PFS without progression and PDS remaining alive.

Figure 4 shows the total 1-year costs for PFS and DPS patients who remained alive throughout the entire year. DPS patients have higher 1-year costs than PFS patients. The mean costs for PFS and DPS patients were approximately $11,566 (95% CI $10,194–$13,153) and $14,519 (95% CI $12,011–$16,871), respectively.

### Monthly Costs

**Figure 5:**
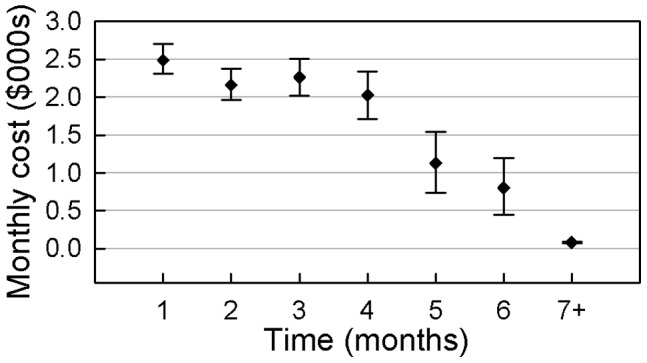
Mean monthly costs (with 95%CI) of PFS without progression.

**Figure 6:**
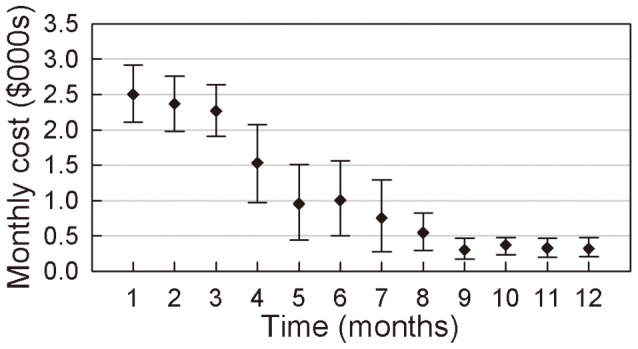
Mean monthly costs (with 95%CI) of PDS remaining alive.

Monthly costs for PFS and DPS patients who remained alive throughout the entire year are presented in Figure 5 and Figure 6. The initial monthly treatment costs for PFS patients (mean $2,490, 95% CI $2,308–$2,702) and DPS patients (mean $2,503, 95% CI $2,110–$2,914) are higher than the mean expenditures in subsequent months.

The mean medical care costs for PFS patients decreased steadily in the first 4 months, and the mean medical care expenditure in the 4^th^ month was $2,024 (95% CI $1,714–$2,335) for this group. A sharp decline in expenditures occurred from the 5th to the 7^th^ months, and costs stabilised after the 7^th^ month. The mean monthly medical care expenditure from the 7^th^ to the 12th month was $82.49 (95% CI $73.90–$91.49). In comparison, the mean medical care costs for DPS patients decreased steadily in the first 3 months, and the mean medical care expenditure by the 3rd month was $2,270 (95% CI $1,910–$2,643). Subsequently, a significant decrease in expenditures occurred in the 4^th^ and 5^th^ months. The mean medical care costs for the 5^th^ and 6^th^ months were $9555 and $1,005, respectively (95% CIs were $442.0–$1,510 and $503.4–$1,558, respectively). The mean monthly costs continued to decline slowly from the 7^th^ to the 9^th^ month and remained steady after the 9^th^ month. The mean medical care costs for the 9^th^ to 12^th^ months were $307.9, $376.2, $331.0, and $326.6, respectively.

### Terminal-phase Costs

**Figure 7:**
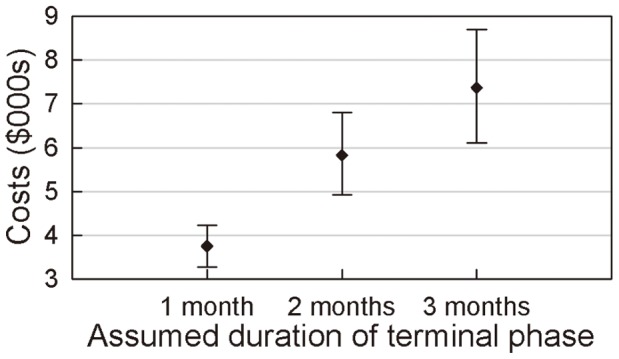
Mean costs (with 95%CI) in terminal phase of 1, 2 and 3 months.

Figure 7 shows the costs associated with end-of-life care provision in patients with advanced NSCLC. The mean medical care costs for the three successive months prior to death were $3,754, $5,829 and $7,372, respectively (95% CIs, $3,274–$4,238, $4,922–$6,807 and $6,109–$8,695, respectively).

## Discussion

This study evaluated the medical costs associated with treating advanced NSCLC in China, estimating the mean cost of care over the 1-year follow-up period for DPS patients ($14,519) to be higher than for PFS patients ($11,566). There are three possible explanations for this result. First, more patients preferred to use tyrosine-kinase inhibitors (TKIs) once their disease progressed, and TKI is more expensive than the standard first-line treatments. Second, patients whose condition progressed further had no recourse but to receive palliative care if TKI failed to halt disease progression, and palliative care is more costly than most active treatments. Last, traditional Chinese medicine was used more frequently in DPS patients because its use was associated with fewer side effects, including weakness, emesis, and anorexia.

For PFS patients, the mean monthly medical care costs remained stable after a slight decline over the first 4 months. Because medications were not adjusted or changed significantly throughout the study period, the fees associated with routine tests and lab work (which are essential in helping doctors to develop rational patient-specific therapy regimens) largely determined any differences in the monthly costs. In addition, responses to chemotherapy were evaluated after every two cycles, so the costs in the third month were slightly higher than were those in the second month. Commonly, four to six cycles of chemotherapy treatment over 3 to 6 months are administered for lung cancer, and most patients do not receive further chemotherapy beyond the 4–6 cycles if they remain in a progression-free state. Any costs associated with the conclusion of chemotherapy were related to adverse reactions to the chemotherapy and routine examinations. Therefore, medical care costs declined sharply after the first 4–6 months. Patients did not undergo further chemotherapy if their condition did not warrant it, but they continued to undergo routine examination every 2 or 3 months. Consequently, monthly medical costs basically stabilised after the 7th month.

For DPS patients, the medical care costs declined slowly in the first 3 months, mirroring the trend observed in the PFS group during the first 4 months. Some patients sought palliative care if their condition did not respond after three treatment cycles, which led to a sharp decrease in the cost of care in the 3^rd^–5th months. In the 6th month, the medical care costs were similar to the 5th month because treatment regimens remained unchanged during these months. In months 6–9, the costs decreased slowly, both because some patients’ condition were under control and because as a benefit of pharmaceutical policy, TKI was free once a patient had received it for 5 months. During the remaining 2 months of the analysis, costs were stable largely because a majority of patients were receiving palliative care at this point.

Medical care costs in the three successive months prior to death were $3,754, $5,829 and $7,372, respectively. During the terminal phase of their disease, patients generally received only supportive care and symptomatic treatment, such as nutrition support, palliative and psychological care, and oftentimes the additional services of rescue and skilled nursing facilities. The additional cost of care in the terminal phase of the disease resulted in medical care expenditures during the last month of life that exceeded any single monthly expenditure for either PFS or DPS patients. Because 17 patients incurred no healthcare costs in their penultimate month of life, the total cost of care during their last 2 months was equal to the monthly expenditure for the 2 months prior to their death. The same situation happened during the last three months for 22 patients. Because the terminal-phase costs were retrospectively calculated from the total costs 3 months prior to death, inevitably, as observed in Figure 7, the mean costs during the terminal phase increased.

Previous studies have reported the medical costs of NSCLC but have lacked a systematic approach for presenting cost data in a format that is suitable for populating cost-effectiveness models. In the United States, Kutikova reported a mean monthly treatment cost of $6,520 per patient (year 2005 value) [19]. The average total management costs in France were estimated to be as high as €39,979±20,279 ($55,526±28,165, year 2007 value) [20]. In Canada, Navaratnam reported that the mean cost per advanced NSCLC patient was $10,805 and the average cost per patient-month ranged from $1,645 to $1,792 in current prices (year 2010 value) [21]. In the Netherlands, the mean total medical cost over 1 year for each patient with advanced NSCLC was €32,840 ($46,914, year 2009 value) [22]. Costs reported in these other studies are higher than the current study. Such differences may occur due to variation in the systems of care provision, financing and reimbursement [23]. Looking for potential factors, we first note that China is a developing country, where costs of medical services and examinations are lower than that in developed countries. It is also important to note that traditional Chinese medicine exerts a high influence on medical care costs in China. Traditional Chinese medicine, used widely throughout China, was integrated into the antineoplastic care strategy in approximately 70% of the cases in this study. A more specific reason may be that as a benefit of pharmaceutical policy in China, TKI was free once a patient had received it for 5 months.

Chouaid et al analysed costs associated with NSCLC in France, estimating separate three monthly costs of US$10,476 for patients receiving first-line treatment, $6,555 for second-line treatment, $539 for remission, and $5764 for the no active treatment phase for patients diagnosed with distant NSCLC [24]. Estimated from 428 patients, the mean aggregate 18 month cost was reported to be $20,184 (95% CI $3,521 to 46,393), which is a slightly higher per month cost than the current study, but the CI is wide. The current study provides cost estimates that are similarly designed to inform cost-effectiveness analyses, but from a Chinese health system perspective, and using alternative categorisation of the disease process, PFS versus DPS over 1 year, 1 month, and during the final 3 months of life.

The current study had two main limitations. First, because this study provides initial estimates, our study population was derived from just one hospital. To comprehensively evaluate the cost of medical care in China, it will be necessary to collect data from more hospitals across the country. Generally, the treatment patterns observed in the sampled NSCLC patients followed NCCN Practice Guidelines in Oncology: Non-Small Cell Lung Cancer (Chinese version) [25]. Thus the difference of treatment patterns between Chinese hospitals is not expected to be significant, especially between upper first-class hospitals. The Second Xiangya Hospital of Central South University is a large-scale, well-equipped (upper first-class) Chinese hospital. In Hunan province, about one third of Lung cancer patients were treated in this hospital. With respect to the unit cost, the cost of majority of drugs in the same geographical area in China is the same. Generally, the cost of drugs account for a large proportion of total Lung cancer treatments, especially for advanced NSCLC. On the basis of this, there is very little difference in unit cost among hospitals in the same geographical area.

With respect to the unit costs, drugs account for a large proportion of total lung cancer treatment costs, and the unit cost for the majority of drugs in the same geographical area in China is the same. Across China more generally, we would also expect only small differences in drug costs as the government attaches great importance to drug price policy, with a strict management system. In addition, there is a drug market price monitoring system in China that strengthens the oversight of drug price, which also acts to stabilize drug costs.

Second, the available data in the hospital may not have been sufficient to assess all of the medical costs from the time of initial diagnosis to death. This lack of data may have influenced our reported results, especially the terminal-phase costs. Any patients who decided to suspend therapy or were transferred to another hospital would have been lost to follow-up. However, in our study only 27 patients were judged to have incomplete data (no medical records in the hospital for more than two months before death), which accounted for 10.7% of all enrolled patients.

This study had two key strengths. First, our data came from a database of real clinical cases, which likely better reflected the true medical care costs for patients. Second, the format of the presented results will be useful for Chinese health economists undertaking further economic analyses, including the use of mathematical modelling in evaluating the cost-effectiveness of treating advanced NSCLC.

Despite these limitations, the costs estimated in this study provide compelling evidence of the significant costs associated with the treatment of advanced NSCLC. The relevance and value of the presented cost data is demonstrated by the frequency with new therapies for the treatment of NSCLC are introduced. In recent years, five prospective randomised clinical trials showed clinical benefits for erlotinib [26], [27] and gefitinib [28]–[30] in EGFR mutation-positive NSCLC, pemetrexed had demonstrated superior general efficacy to a range of comparators [31]–[34], and in patients with advanced NSCLC, rh-endostatin plus platinum-based chemotherapy has shown significantly longer PFS [35], [36]. All of these new treatments are more costly than the therapies they are designed to replace, and so cost-effectiveness analyses are required to make funding decisions. Unfortunately, costs associated with those treatments are often difficult to estimate due to a lack of available and current data, especially in China, where the development of pharmacoeconomics is slow. The costing data provided by our study will be of great value to these cost-effectiveness analyses.

In conclusion, the economic evaluation of health care technologies is becoming ever more important in China, especially in disease areas for which new and expensive therapies are being introduced on a regular basis, such as NSCLC. This is first paper to present empirically estimated China-specific costs associated with the treatment of NSCLC by disease stage, in a format that is specifically intended to inform cost-effectiveness analyses of treatments for NSCLC, and hence, contribute to the more efficient allocation of limited healthcare resources in China.
